# P-79. Racial/ethnic disparities and facility-level variation in major lower limb amputation among patients with a new diagnosis of diabetic foot ulcer within the Veterans Health Administration

**DOI:** 10.1093/ofid/ofae631.286

**Published:** 2025-01-29

**Authors:** Hiroyuki Suzuki, Mary Vaughan-Sarrazin, Michael Ohl, Bradley Mecham, Kimberly Mccoy, Daniel J Livorsi

**Affiliations:** University of Iowa Carver College of Medicine, Iowa City, Iowa; University of Iowa, Iowa City, Iowa; University of Iowa Carver College of Medicine, Iowa City, Iowa; Iowa City VA, Iowa City, Iowa; Iowa City VA, Iowa City, Iowa; University of Iowa Carver College of Medicine, Iowa City, Iowa

## Abstract

**Background:**

Diabetic foot ulcers (DFU) are associated with high morbidity and mortality, including major lower limb amputation (LLA). Little is known about racial/ethnic disparities and facility level variation in major LLA in large healthcare systems, such as Veterans Health Administration (VHA). VHA cares for over 9 million Veterans in 140 facilities with no insurance-related barriers to care.

**Figure:**
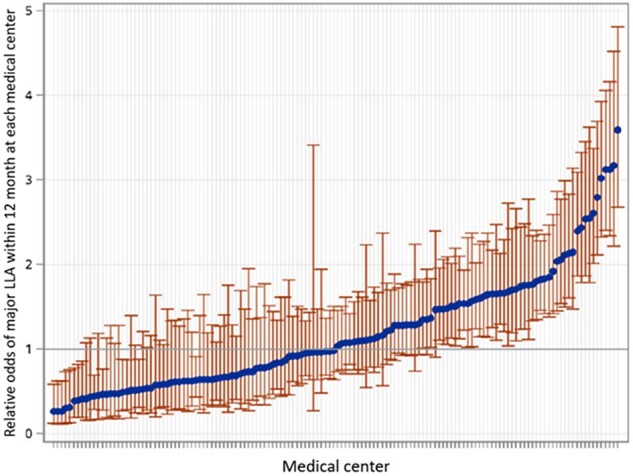
Caterpillar plot of relative odds of major LLA in each medical center

**Methods:**

This is a retrospective cohort study of all Veterans with a new diagnosis of DFU during 2016-2021. The outcome was major LLA within 12 months from DFU diagnosis. We conducted multivariable logistic regression with random facility intercepts to assess variation in major LLA across VHA facilities while adjusting for patient demographics, comorbidities, and the severity of DFU at initial diagnosis. We calculated odds ratios (ORs) and confidence intervals (CIs) for associations between patient-level variables and major LLA. We calculated the median OR (MOR) as a measure of facility-level variation in major LLA. The MOR can be interpreted as the median change in odds of major LLA associated with care in all pairs of facilities.

**Results:**

There were 105,644 patients from 140 hospitals in the cohort. The median age was 67. Most patients were White (75.6%), followed by Black (18.5%) and Hispanic (6.0%). At presentation, 91.6% had an early-stage ulcer while the rest had complicated DFU. Major LLA occurred in 4055 patients (3.8%). In the logistic regression model, variables associated with higher odds of major LLA were Black (OR 1.77, 95%CI 1.62-1.92), Native American (OR 2.08, 95%CI 1.57-2.75), Hispanic (OR 1.38, 95%CI 1.19-1.60), complicated DFU (OR 3.36, 95%CI 3.11-3.64), chronic kidney disease (OR 1.11, 95%CI 1.01-1.21), peripheral vascular disease (OR 3.49, 95%CI 3.24-3.76) and myocardial infarction (OR 1.32, 95%CI 1.21-1.49). The facility-level variation in major LLA (MOR: 1.87, p< 0.001) was larger than most of patient-level risk factors, suggesting that facility-level factors play an important role in the outcome of DFU.

**Conclusion:**

Our study showed racial/ethnic disparities and facility-level variation in major LLA within the VHA. Future studies should investigate the reasons for this variation and develop strategies for reducing disparities in LLA.

**Disclosures:**

**Daniel J. Livorsi, MD**, Merck: Grant/Research Support

